# Interventions for supporting nurse retention in rural and remote areas: an umbrella review

**DOI:** 10.1186/1478-4491-11-44

**Published:** 2013-09-11

**Authors:** Gisèle Mbemba, Marie-Pierre Gagnon, Guy Paré, José Côté

**Affiliations:** 1 Hôpital St-François d’Assise, Research Center of the Centre Hospitalier Universitaire de Québec, 10 rue de l’Espinay, D6-727, G1L 3L5, Québec, QC, Canada; 2 Department of Information Technology, 3000, chemin de la Côte-Sainte-Catherine, HEC Montréal, H3T 2A7, Montréal, QC, Canada; 3 Faculty of Nursing Sciences, Pavillon Marguerite-d'Youville, C.P. 6128 succ. Centre-ville Université de Montréal, H3C 3J7, Montréal, QC, Canada

**Keywords:** Nurse shortage, Nurse retention, Rural retention, Rural health services, Umbrella review

## Abstract

**Context:**

Retention of nursing staff is a growing concern in many countries, especially in rural, remote or isolated regions, where it has major consequences on the accessibility of health services.

**Purpose:**

This umbrella review aims to synthesize the current evidence on the effectiveness of interventions to promote nurse retention in rural or remote areas, and to present a taxonomy of potential strategies to improve nurse retention in those regions.

**Methods:**

We conducted an overview of systematic reviews, including the following steps: exploring scientific literature through predetermined criteria and extracting relevant information by two independents reviewers. We used the PRISMA (Preferred Reporting Items for Systematic reviews and Meta-Analyses) criteria in order to assess the quality of the reports.

**Findings:**

Of 517 screened publications, we included five reviews. Two reviews showed that financial-incentive programs have substantial evidence to improve the distribution of human resources for health. The other three reviews highlighted supportive relationships in nursing, information and communication technologies support and rural health career pathways as factors influencing nurse retention in rural and remote areas. Overall, the quality of the reviews was acceptable.

**Conclusions:**

This overview provides a guide to orient future rural and remote nurse retention interventions. We distinguish four broad types of interventions: education and continuous professional development interventions, regulatory interventions, financial incentives, and personal and professional support. More knowledge is needed regarding the effectiveness of specific strategies to address the factors known to contribute to nurse retention in rural and remote areas. In order to ensure knowledge translation, retention strategies should be rigorously evaluated using appropriate designs.

## Introduction

Understanding nursing shortage on a global scale is a complex matter. The definition or measure of nurse shortage [1,2] or the establishment of the right ratio of nurses to population [1] may vary between countries and make statistical comparisons harder. However, as Buchan and Aiken pointed out, nursing shortage “is not just about number, but about how the health system functions to enable nurses to use their skills effectively” [1]. When nursing shortage occurs, on the local or global scale, it may lead, if not addressed, to the failure of healthcare [1].

Acute nursing shortage is a growing concern in developing and developed countries [1,2]. In a survey among 70 members states of the World Health Organization (WHO), nursing shortage was experienced by 86% of them and, in 54% of these countries, nursing shortage was of a great intensity [3]. In a developed country such as Canada, recruiting and retaining nurses is becoming a main challenge for decision makers [4] in the light of the predicted nursing shortage and its concomitant effects on healthcare [5]. There is a current shortfall of 22,000 nurses, and a shortfall of 60,000 full-time equivalent nurses is expected by 2022 [6].

The literature mentions close ties between nursing shortage and geographical imbalances in nursing [7,8] or the health workforce [9-12]. Some studies focus on international nurse migration and the ‘brain drain’ from developing to developed countries [2]. Other studies stress the remote, rural and underserved areas where nursing shortage is more acute [13], both in developing [8,14] and developed countries [7,15].

Rural and remote areas have more difficulties in recruiting and retaining nurses when compared to the greater career opportunities and work prospects in urban areas [1,7,12]. Additional barriers to recruitment and retention of healthcare staff in remote areas are poor working conditions [9,16], professional isolation [17], lack of services in the general living environment [16], and higher mobility of health professionals associated with globalization [9]. Insufficient numbers and loss of nurses and the health workforce impede rural and remote populations to obtain healthcare services. As reported by the WHO, only 38% of the nursing workforce remains in rural areas, where almost half of the world’s population lives [13].

The evidence about the effectiveness of rural retention interventions comes mostly from advanced economies like Australia, Canada or the USA [10]. Among studies of nurse or health worker retention in remote areas from developed countries, the rural background [7,10,15,18] or the rural integration [18,19] may constitute a powerful predictor of rural practice. These personal and social factors are interconnected with others that influence the decision of nurses or health workers to stay in or leave rural and remote areas: financial aspects, career aspirations, working and living conditions and bounding or mandatory service [11,13]. In order to have a better understanding of the factors influencing the retention of health staff, the WHO has elaborated a model of heath workers’ decision to relocate, stay, or leave rural and remote areas that proposes four categories of interventions to improve their retention in these settings: education, regulation, financial incentives, and personal and professional support [13].

The objective of this review is to synthesize the current scientific evidence on interventions to promote nurse retention in rural, peripheral or remote areas, and to present a taxonomy of potential strategies in order to propose further research directions. The rest of this paper is organized as follows: the first section outlines the research methodology used for this review; the second section presents the results of research about interventions for supporting nurse retention in rural and remote areas; and the third section proposes a taxonomy of nurse retention strategies, based on the WHO model, as well as potential future research propositions.

## Methods

We performed a systematic review of prior reviews, a method also known as umbrella review [20-22], to synthesize the scientific evidence regarding interventions that support nurse retention in rural, peripheral or remote areas. We used the following keywords, and their variations, in combination with each other: nurse shortage, nurse retention, rural retention, systematic review or literature review. We consulted the following databases: MEDLINE (PubMed interface), CINAHL, EMBASE and the search engine Google Scholar. While the search was international, we limited inclusion of publications from a 22-year period (January 1 1990 to July 31 2012). The included studies met the following inclusion criteria: (i) derived from a systematic review; (ii) involved nursing professionals; (iii) assessed factors that influenced retention in rural or remote areas; and (iv) were published in English, French, or Spanish. We excluded studies that were not reviews, did not involve nurses, did not specifically concern rural and remote areas, and were published in other languages.

**Figure 1:**
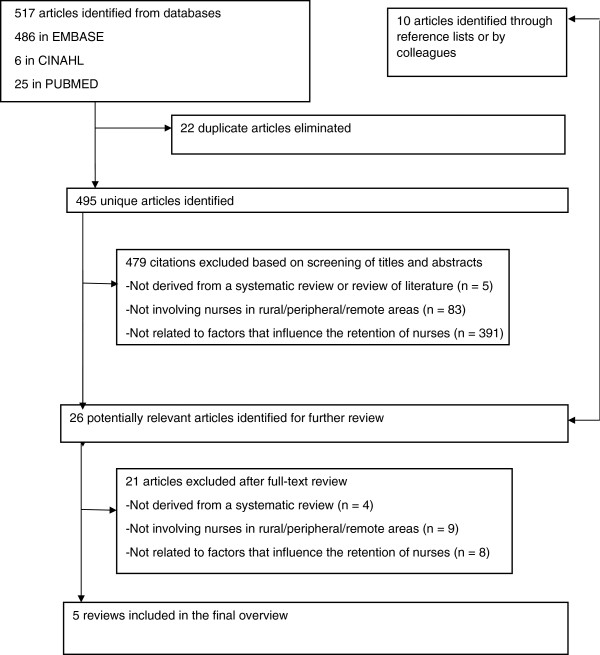
Study selection flow.

The systematic review process is shown in Figure 1. First, two members of the research team (GM and MPG) independently read the title and abstract of each retrieved article to identify potentially relevant reviews. Then, the same two persons independently reviewed the full text of each potentially relevant article, compared their results and agreed about the final codification. Finally, we used the Preferred Reporting Items for Systematic reviews and Meta-Analyses (PRISMA) criteria for reporting systematic reviews and meta-analyses of studies that evaluate healthcare interventions, a quality appraisal tool developed by Liberati and colleagues [23]. This evaluation of the quality of reporting did not aim to decide study inclusion or exclusion, but rather to consider this score in the interpretation of our results.

## Results

From an initial pool of 517 publications, we selected a total of five review articles that met the inclusion criteria [24-28].

Table 1 presents the five reviews of nurse retention interventions in rural and remote areas that were selected. The first one examined the effects of financial incentives for return of service in underserved areas [24]. The second synthesized the available evidence regarding the effectiveness of retention strategies for health workers in rural and remote areas [25]. The third review defined the three main supportive relationships identified in the nursing literature [26]. The fourth one explored the impact of interventions using information and communication technologies (ICTs) on recruitment and retention of healthcare professionals in less-served regions [27], while the fifth review described stages related to recruitment and retention of health professionals to rural health careers [28]. As shown in Table 1, three of the reviews [24,25,27] met more than 66% of the PRISMA appraisal criteria while the other two [26,28] met more than 50% of them.

**Table 1:** Profile of the reviews considered in this overview

**Title**	**Authors**	**Year of publication**	**Country**	**Number of included studies**	**Type of professionals**	**Study variables**	**PRISMA score**
Financial incentives for return of service in underserved areas: a systematic review	Bärnighausen and Bloom [24]	2009	USA	43	Nurses and physicians	Financial incentives	22/27
Systematic review of effective retention incentives for health workers in rural and remote areas: towards evidence-based policy	Buykx *et al*. [25]	2010	Australia	14	Nurses and physicians	Retention strategies (financial incentives, loan repayment)	19/27
Mentoring, clinical supervision and preceptoring: clarifying the conceptual definitions for Australian rural nurses: a review of the literature	Mills *et al*. [26]	2005	Australia	Not specified	Nurses	Supportive relationships in nursing: mentoring, clinical supervision and preceptoring	17/27
Supporting health professionals through information and communication technologies: a systematic review of the effects of information and communication technologies on recruitment and retention	Gagnon *et al*. [27]	2011	Canada	13	Nurses and other care providers	ICTs support	21/27
Rural health career pathways: research themes in recruitment and retention	Fisher and Fraser [28]	2010	Australia	Not specified	Nurses and other providers	Stages of rural career pathways	17/27

*ICTs* information and communication technologies, *PRISMA* Preferred Reporting Items for Systematic reviews and Meta-Analyses.

From the profile of the reviews included in this study, we have classified interventions into four different themes: financial incentives, supportive relationships in nursing, ICT support, and career pathways for rural health.

### Financial incentives

The review by Bärnighausen and Bloom [24] synthesized 43 empirical studies, 34 of which investigated financial incentives in the USA. The remaining studies evaluated programs in Japan (five studies), Canada (two), New Zealand (one) and South Africa (one). The review identified five different types of financial-incentive programs for return of service: service requiring scholarships, educational loans with service requirements, service-option educational loans, loan repayment programs, and direct financial incentives. Direct financial incentives are usually provided at the entry into practice in an underserved area and the money from this type of program can be used for different purposes, whereas loan repayments are provided after each period of service, and the money must be spent on healthcare education. Studies included in the review by Bärnighausen and Bloom [24] contributed to several outcomes such as: 1) program outcomes among participants which encompass recruitment (14 studies), retention (17 studies), participant satisfaction (7 studies), and family satisfaction (3 studies); 2) program effectiveness at the individual level with outcomes on provision of care (11 studies), retention (7 studies), and participant satisfaction (2 studies); 3) program effectiveness at the population level with outcomes on health system (6 studies), and health outcomes (for example, mortality) (1 study). The number of studies in parentheses adds up to 68, because some studies contribute two or three outcomes each.

Globally, this systematic review found substantial evidence of the effectiveness of financial-incentive programs for return of service as a health policy intervention to attract human health resources in underserved areas. Evidence on the impact of financial incentives was, however, limited regarding retention in rural areas. Limitations of this review include the fact that a majority of included studies were from the USA and only one study took place in a low-resource country (South Africa). Furthermore, the number of studies that included nurses is not provided. The results from this review show that financial-incentive programs placed substantial numbers of health workers in underserved areas and that program participants were more likely than non-participants to work in underserved areas in the long run, even though they were less likely to remain at their site of original placement. In terms of quality of reporting, the review met more than 66% of the PRISMA criteria.

Next, the systematic review by Buykx and colleagues synthesized the effectiveness of retention strategies for health workers in rural and remote areas [25]. However, this review of 14 papers found only one study on the effectiveness of nurse retention strategy. Six studies focused on medical practitioners, five were about healthcare professionals in general but with strong emphasis on medical doctors, and one focused on psychiatrists.

Financial incentives were the most commonly reported retention strategies. This systematic review provides limited support to the effectiveness of financial incentive interventions, suggesting that financial incentives are more effective to improve recruitment and short-term retention of healthcare workers than for fostering their long-term retention in underserved areas. Strategies in which health workers have some form of obligation (such as visa conditions restricting area of practice or loan repayment) could be more effective in retaining them on a longer period. However, there is some evidence that indicates that non-financial incentives, such as providing quality working and housing conditions, could have more impact on the decision of healthcare workers to stay in the area.

### Supportive relationships in nursing

The review by Mills and colleagues [26] examined the three main supportive relationships identified in the nursing literature that affect retention and recruitment: mentoring, clinical supervision and preceptoring. This review aimed to highlight the similarities and differences among them and to illuminate the range of possible supportive relationships that could be fostered by decision-makers. Each of these three types of coaching will be examined in turn.

#### Mentoring

According to Steward and Krueger [29], the definition that most adequately reflects the concept of mentoring in nursing today is “Mentoring in nursing is a teaching-learning process acquired through personal experience within a one-to-one, reciprocal, career development relationship between two individuals diverse in age, personality, life cycle, professional status, and/or credentials” (page 315).

This systematic review [26] identified very few studies that explicitly focused on rural nurse mentors. One study was from the USA and explored the outcomes of mentoring partnerships arranged between academic mentors and beginner rural nurse practitioners. However, there are formal mentoring programs in Australia that are also mentioned [26]. One of these programs involved undergraduate rural and remote nurses, aged care nurses and re-entry to practice nurses who held Australian government scholarships. This comprehensive evaluation of a mentoring project, initiated by the Association for Australian Rural Nurses, has highlighted the influence of continuing education on rural nurse mentors and the relationships that they form with their mentees [26].

#### Clinical supervision

The authors of this review [26] defined clinical supervision as “a support mechanism for practising professionals within which they can share clinical, organisational, development and emotional experiences with another professional in a secure confidential environment in order to enhance knowledge and skills” (page 4). This process will lead to an increased awareness of other concepts including accountability and reflective practice [30]. One-on-one, triad, and group represent three forms of clinical supervision. According to this systematic review [26], group clinical supervision would be particularly effective, especially if conducted off-site, held frequently and of substantial length. Also, the rural nurses felt that their clinical supervision experiences were valuable in improving their understanding about their practice, as well as increasing their self-awareness and ability to critically reflect [31].

#### Preceptoring

Unlike mentoring and clinical supervision, preceptoring in nursing involves clinical staff, as opposed to faculty staff, in order to provide supervision and clinical instruction to undergraduate or newly registered nurses, or those new to a specific clinical environment [32]. Usually, preceptoring is conducted on a one-on-one basis. This approach strengthens the relationship between the undergraduate or newly registered nurse and their preceptor to quickly adapt in the workplace. Also, the development of a preceptorship program is a way to bridge the gap between nursing education and service [32]. However, the ‘clinical teaching associate’ is another model of preceptoring where healthcare facilities are funded by university in order to release a clinician, often called the clinical associate, who is responsible for supervising and teaching a small group of students [33].

In conclusion, Australian rural nursing experiences showed that mentoring, clinical supervision, and preceptoring are all valuable in meeting the particular challenge of recruitment and retention of rural nurses. Thus, these strategies seem to be essential when considering policies aiming at ensuring recruitment and retention of rural nurses in the future. One strength of this review is that it focuses only on studies involving nurses. In terms of rigor, the review met more than 50% of the critical appraisal criteria.

### Information and communication technologies support

In their systematic review, Gagnon and colleagues [27] synthesized 13 studies in order to explore the impact of interventions using ICTs on recruitment and retention of healthcare professionals. Of the 13 studies, five are related to both recruitment and retention. Five other studies exclusively examine retention, whereas the other three pertain only to recruitment. Except for one older study, all other studies target the domain of telehealth. This review showed that ICTs might have positive effects on the recruitment and retention of healthcare professionals in rural and remote regions. For instance, one study showed the influence of telehealth on the decision of surveyed physicians to stay in rural practice [34], and another showed the positive impact of telehealth on nurse retention [35].

However, it seems that the effects of ICTs are more noticeable on the diverse constitutive recruitment and retention factors, such as reduction of professional isolation, networking, decision-making support, improvement of quality of life, and job satisfaction. Although very few studies have investigated this topic, it is estimated that these results can be in part transferable to the situation of nurses who practice in remote areas. In terms of rigor, this review met more than 66% of the PRISMA quality appraisal criteria.

### Career pathways for rural health

The review by Fisher and Fraser [28] identified four stages of rural career pathways. These authors have consulted the research literature on recruitment and retention to rural health careers (principally in developed regions such as Australia, New Zealand, Europe, the USA and Canada) and propose a framework consisting of four stages that is similar to the ‘rural pipeline’ of physicians described in the medical literature. In this review, the notion of the pipeline is broadened. It embraces other health professions, especially nursing.

The different stages are described as follows. The first stage, 'making career choices' (structured contact between secondary schools and health professionals), includes promotion of health careers such as medicine, nursing and allied health [36].

The second stage, 'being attached to place' (rural student selection), concerns attraction of rural students. The literature supports the relationship between attachment to the place where the student originates and rural practice, thus favoring the selection of rural students into medical programs in Australia [37]. Moreover, long-term living in a rural community could also contribute to this attachment by increasing social bonds among members of the community [38]. However, information available on the effects of rural student selection in professions such as allied health and nursing remains low.

The third stage, 'taking up rural practice' (rural exposure), acknowledges that exposure to rural clinical settings and different locations can increase interest in rural practice for medical, nursing and allied health students [39]. The authors concluded that rural exposure could indeed increase interest in rural practice, but that cultural issues are essential to consider in the rural nursing profession to improve the nurturing role that supervisors provide to students.

The fourth stage, 'remaining in rural practice' (educational and professional support), is concerned with time spent in a rural context, contentment of rural life and balancing personal and professional roles. Some important factors influencing the retention of rural health professionals are identified; for instance, nurses identified professional support as an important component of retaining nursing staff in rural areas.

Overall, several studies in this systematic review suggest that both personal and work-related factors can impact retention, which requires strategies that address these multiple causes simultaneously. The ‘rural pipeline’ model could be a useful template for future research because it describes the various phases in the rural practice for which specific interventions may be more effective. Using a common framework would also enhance and consolidate evidence to ensure a coordinated approach to recruitment and retention of all health professionals in rural areas. In terms of rigor, this review scored lower because it lacks information about the number of included studies and the type of health professionals who are targeted in each study.

### Taxonomy of nurse retention interventions in rural and remote areas

In this section, we propose a taxonomy of strategies to increase nurse retention in rural and remote areas. These interventions are extracted from the systematic reviews described above. The strength of evidence associated to each intervention is also provided, based on review authors’ conclusions regarding the effectiveness of each specific intervention and also on the fact that this evidence concerns nurses (direct evidence) or any healthcare providers (indirect evidence). Table 2 presents the specific interventions assessed in the five included reviews, according to the four broad types of interventions proposed in the WHO model [13]: (i) education and continuous professional development interventions, (ii) regulatory interventions, (iii) financial incentives (direct and indirect), and (iv) personal and professional support.

**Table 2:** Taxonomy of nurse retention interventions in rural and remote areas

**Category of intervention**	**Effective interventions for nurse retention**	Strength of evidence^*^
A. Education and continuous professional development interventions	-Recruitment from, and training in, rural areas	Moderate [26]^#^
	-Targeted admission of students from rural background	Moderate [28]^#^
	-Early and increased exposure to rural practice during undergraduate studies	
	-Support for continuous professional development	
B. Regulatory interventions	-Increased opportunities for recruitment to civil service	Low [25]^#^
	-Recognizing overseas qualifications	
	-Producing different types of health workers	
C. Financial incentives	-Direct and indirect financial incentives (direct payments, service-requiring scholarships, educational loans with service requirements, loan repayment programs)	Moderate [24]^#^
		Low [25]
D. Personal and professional support	-General improvement in rural infrastructure (housing, roads, phones, water supplies, radio communication, etc.)	Strong [25]
	-Supportive supervision (mentorship, preceptorship, clinical supervision)	Moderate [26]^#^
	-Measures to reduce health workers’ feeling of isolation (professional/specialist networks, telemedicine and telehealth)	Moderate [27]^#^

^*^Strength of evidence based on review authors’ conclusions.

^#^Indirect evidence: the original studies on which authors based their conclusions are not specific to nurse retention.

#### Education and continuous professional interventions

Education and continuous professional interventions encompass several activities, such as making career choices. These interventions include promotion of careers such as medicine, nursing and allied health. According to Fisher and Fraser [28], these approaches could overlook some of the socioeconomic barriers that rural students may face when making their career choice. Rural exposure has some positive outcomes in increasing interest in rural practice for medical, nursing and allied health students. Access to educational opportunities is an important factor contributing to nurse retention in remote and rural areas. Although there is a well-developed program of continuing education for medical practitioners, it seems that continuing education programs for other health professionals are less developed. Current evidence suggests that the coordination of a structured support system could strengthen and sustain retention of health professionals in rural areas [40]. However, ICTs can have an influence on factors related to the recruitment and retention, but the impact of ICTs on nurse retention has received little attention in the scientific literature. Some studies indicate that these technologies could support nurse retention through improved professional development [27].

#### Regulatory interventions

Regulatory interventions to support recruitment and retention in rural areas are related to expanding the scope of practice of rural health workers, and developing new categories of health workers. Foreign-recognition of qualifications represents another regulatory measure but it mostly applies to physicians. Also, little evidence is available regarding facilitated entry into universities for rural students in allied health and nursing professions [27].

#### Financial incentives

Financial incentives are the only strategies that have been largely evaluated. However, evidence of their effectiveness to support nurse retention in remote and rural areas remains limited as of today. For instance, Buykx and colleagues [25] consider that financial incentives are not very effective for long-term retention.

#### Personal and professional support

Personal and professional support creates a supportive relationship for nurses contemplating a shift to the rural environment at the beginning of their employment and may provide an incentive to stay. Evidence from the review by Mills and colleagues [26] indicate that mentoring, clinical supervision and preceptoring are key measures that needs to be factored into rural health service planning for undergraduate students contemplating a rural nursing career. For practicing rural nurses, supportive relationships within the workplace represent a pragmatic commitment that entices them to stay.

## Discussion and conclusion

This umbrella review reveals that financial incentives, supportive relationships in nursing (mentoring, clinical, supervision, preceptoring), ICT support and the career pathways for rural health constitute potential strategies that could influence the retention of nurses in rural and remote areas. Even though the impact of financial interventions is supported by two reviews [24,25], most of the studies included in these reviews are from the USA, making the results less applicable to countries with a different healthcare system such as Australia and Canada, or to low- and middle-income countries.

Very few empirical studies explored the impact of supportive relationships in nursing and, thus, the evidence regarding this type of intervention is quite limited. The review on ICT usage found some evidence of its effect on medical professionals; however, there is a lack of literature on other health workers [27]. Although research specific to rural nursing is growing, it is still very limited [41].

This umbrella review aimed to synthesize the main factors that influence nurse retention in rural or remote areas. This synthesis allows us to propose a taxonomy of interventions for rural and remote nurse retention. This taxonomy is based on the categories of the model proposed by the WHO. It is also inspired by the framework proposed by Buykx and colleagues [25]. In order to maximize the use of retention funding for the purpose of minimizing avoidable turnover, Buykx and colleagues [25] suggest using a framework that addresses known determinants of poor retention which could support resource allocation decisions based on scientific evidence. Retention funding should also be seen as an ingredient of a comprehensive retention strategy developed on the basis of local needs. Finally, Buykx and colleagues suggest that the impact of strategies on subsequent workforce retention should be assessed using rigorous evaluation methods [25]. Given the limited empirical evidence available on effective retention strategies for rural and remote nurses, we also encourage more research on the main determinants of nursing retention in rural and remote areas that could inform future strategies.

### Limitations

This umbrella review provides a starting point in order to orient future interventions aiming at increasing nurse retention in rural and remote areas. However, results should be interpreted with caution as we did not look at individual studies included in the five systematic reviews. Thus, our interpretation relies on the judgment of review authors. Also, the average PRISMA score of some reviews mean that the results should be interpreted with caution. Although we tried to include reviews of interventions for nurse retention in rural or remote areas, most of the reviews also included other health providers, highlighting the need for more reviews focusing on retention strategies specific to nursing.

### Final remarks/need for further research

The use of the PRISMA checklist of items for reporting systematic reviews showed that only two-thirds of items are respected in most of the studies. Items such as existence of a protocol and registration, risk of bias in individual studies and across studies, study characteristics, additional analyses and funding are not often mentioned. We suggest that authors of future systematic reviews draw upon the PRISMA checklist to report their methodology and results.

## Abbreviations

ICTs: Information and communication technologies; PRISMA: Preferred reporting items for systematic reviews and meta-analyses; WHO: World Health Organization.

## Competing interests

The authors declare that they have no competing interests.

## Authors’ contributions

GM and MPG reviewed abstracts for the umbrella review and drafted the manuscript. GP and JC revised and contributed to the manuscript. All authors read and approved the final manuscript.
